# Neural responses to morally laden interactions in female inmates with psychopathy

**DOI:** 10.1016/j.nicl.2021.102645

**Published:** 2021-03-27

**Authors:** Keith J. Yoder, Carla Harenski, Kent A. Kiehl, Jean Decety

**Affiliations:** a Department of Psychology, University of Chicago, Chicago, IL, USA; b The Mind Research Network and Lovelace Biomedical, Albuquerque, NM, USA; c Department of Psychology, University of New Mexico, Albuquerque, NM, USA; d Department of Psychiatry, and Behavioral Neuroscience, University of Chicago, Chicago, IL, USA

**Keywords:** Emotion understanding, Decision-making, Empathy, Functional connectivity, Functional MRI, Forensic neuroscience - Moral evaluation, Female Psychopathy

## Abstract

Much of social cognition requires making inferences about the mental and emotional states of others. Moreover, understanding the emotions of others is an important foundation for moral decision-making. Psychopathy is associated with both aberrant emotional understanding and atypical hemodynamic responses when viewing and evaluating morally laden social interactions. In the present functional MRI study, female inmates (N = 107) were asked to evaluate the likely emotional state of either the recipient or the initiator of harmful or helpful interactions. Psychopathy was assessed with the Psychopathy Checklist-Revised (PCL-R). PCL-R scores were not associated with differences in confidence or accuracy ratings. However, psychopathy scores were significantly related to increased hemodynamic response in right dorsolateral prefrontal cortex when viewing harmful interactions and decreased functional connectivity from right amygdala to inferior parietal cortex and insula, and from temporal parietal junction to dorsomedial prefrontal cortex. Overall, this work indicates that in females, psychopathy is associated with normal behavioral accuracy and confidence but alterations in neural network activity during moral decision-making.

## Highlights

• Psychopathic personality traits do not predict emotional accuracy in female inmates.
• When viewing harmful interactions, PCL-R scores are associated with dlPFC response.
• PCL-R scores are linked to lower functional connectivity seeded in amygdala and TPJ.

## 1 Introduction

Humans, as social creatures, readily attribute emotional and cognitive states to others when engaging in or observing third-party social interactions. Emotion is an adaptive orienting system that evolved to guide behavior. Emotion is also an interpersonal communication system that elicits response from others. Thus, emotions can be viewed both as intrapersonal and interpersonal states (Decety and Skelly, 2014). Cognitive (e.g., attentional processes) and emotional processes are not entirely separate entities. Rather, signals interact and are integrated at both perceptual and executive levels (Pessoa, 2014).

Psychopathy is a personality disorder which includes a constellation of traits, such as dishonesty, superficial charm, lack of empathy or guilt, and impulsive behavior (Cleckly, 1941). Importantly, dysfunctional socioemotional processing is a core feature of psychopathy (Hare, 2016, Kiehl, 2015), and atypical emotional processing, such as deficits in empathy, is a risk factor for violent or criminal behavior (Anderson and Kiehl, 2012, Blair, 2008, Olver and Wong, 2015, Seara-Cardoso and Viding, 2014). Conflicting behavioral and neuroscience investigations have led to ongoing debates about the specificity of atypical empathic processing, particularly whether deficits are limited to distinct emotions (e.g. fear and/or sadness) or are more general (Book et al., 2007, Dawel et al., 2012, Deming et al., 2020, Glass and Newman, 2006, Hastings et al., 2008, Marsh and Blair, 2008, Seara-Cardoso et al., 2012). While both cognitive and emotional deficits have been documented in individuals with high levels of psychopathic traits (Anderson et al., 2017), most neuroscience studies report atypical neural responses during perception and recognition of the emotions of others (Decety et al., 2015, Decety et al., 2013b, Deming et al., 2020, Marsh et al., 2013, Sato et al., 2011). Further, two recent meta-analyses indicate that across a variety of tasks, psychopathy is marked by atypical neural responses in prefrontal, paralimbic, and insular regions (Deming and Koenigs, 2020, Poeppl et al., 2019).

Socioemotional processing plays a critical role in moral reasoning (Haidt and Graham, 2007, Van Bavel et al., 2015). Notably, harm aversion is a crucial component of moral cognition (Decety and Cowell, 2018, Gray et al., 2012, Miller and Cushman, 2013). Studies reporting moral insensitivity in individuals with psychopathy often interpret these effects as stemming from lack of empathy and callous disregard for others (Blair, 2007, Cheng et al., 2012, Decety and Cowell, 2014a, Harenski and Kiehl, 2011, Lockwood, 2016). Using the Moral Foundations framework, which articulates distinct moral domains (Graham et al., 2011), psychopathy was originally linked to specific reductions in concern for the domains of harm and fairness (Aharoni et al., 2011, Glenn et al., 2009a), though more recent work has identified reduced concern across all domains (Jonason et al., 2015). Conversely, research utilizing sacrificial moral dilemmas has produced conflicting results, with some studies finding associations between psychopathic traits and greater endorsement of utilitarian judgments among both undergraduates and incarcerated populations (Bartels and Pizarro, 2011, Koenigs et al., 2012), while others find no differences in behavioral responses (Cima et al., 2010, Glenn et al., 2009a, Tassy et al., 2013). A recent meta-analysis concluded that psychopathy is weakly associated with abnormal moral decision-making, rather than being characterized by pronounced or overarching moral deficits (Marshall et al., 2018). A complementary motivational account suggests that psychopathy is associated with relatively intact moral understanding but a lack of motivation to apply this moral knowledge (Cima et al., 2010, Glenn et al., 2009a, Tassy et al., 2013). Moreover, a growing body of neuroscience evidence suggests that when individuals with psychopathy provide moral evaluations that are indistinguishable from controls, as is often the case, they do so by recruiting different brain circuits (Aharoni et al., 2012, Yoder et al., 2015a).

However, the vast majority of studies examining neural functioning in psychopathy have included only male participants. Psychopathy is a well-documented risk factor for violent behavior and criminality in males (Blais et al., 2014, Guy et al., 2010), and predicts violent recidivism and future violence in prison (Camp et al., 2013, Olver and Wong, 2015). Emerging evidence from adolescents with conduct disorder suggests that there are important sex differences in the impact of psychopathic traits on brain structure and function (Michalska et al., 2015, Smaragdi et al., 2017). The rates of female incarceration have risen over the last decade (Carson and Anderson, 2016), indicating the pressing importance of investigating psychopathic traits in female offenders and determining whether the established links between psychopathic traits and abnormal neural functioning observed in men also manifest in women.

There is some debate about the specificity of empathic deficits in psychopathy. Empathy is a multifaceted construct which includes affective, cognitive and motivational facets (Decety and Jackson, 2004, Lockwood, 2016, Shamay-Tsoory, 2009), and each facet seems uniquely related to moral cognition (Decety and Cowell, 2014b). Affective empathy includes both the tendency to experience emotional distress in response to the distress of others, as well as a motivation to respond appropriately to another person’s emotional state. Cognitive empathy refers to an individual’s propensity to adopt the perspective of another person and imagine what another person is thinking or feeling (Shamay-Tsoory, 2009). Cognitive empathy is closely related to theory of mind, the ability to infer the beliefs and intentions of others as separate from oneself (Decety and Jackson, 2004). Though some have argued that sex differences in empathy are fundamental to psychological differences (Baron-Cohen and Wheelwright, 2004), decades of research indicates that sex differences in empathy are large when measured using self-reports, but quickly diminish, or even become nonexistent, when using behavioral measures and functional neuroimaging methods, such as responding to others’ pain (Baez et al., 2017, Eisenberg and Lennon, 1983, Michalska et al., 2013). Some evidence suggests that psychopathy is associated with reduced accuracy when inferring the emotional states of others (Brook et al., 2013, Brook and Kosson, 2013), though the impact of psychopathic traits on emotion processing does not always replicate in women (e.g., Vitale et al., 2011). Other work indicates high levels of psychopathic traits are associated with deficits in inferring affective states (e.g. emotions) alongside an intact ability to infer cognitive states (e.g. beliefs) of others (Shamay-Tsoory et al., 2010). Converging evidence from functional neuroimaging and lesion studies indicate that the temporoparietal junction (TPJ), which is situated at the posterior superior temporal sulcus (pSTS), and medial prefrontal cortex (mPFC) are essential for detecting and representing mental states of others, and play an important role in cognitive empathy and moral cognition (Decety and Lamm, 2007, Gallagher and Frith, 2003, Lamm et al., 2007, Moll et al., 2007, Saxe et al., 2004, Silani et al., 2013, Yoder and Decety, 2014a). In healthy participants, viewing others in pain reliably elicits response in dorsal anterior cingulate (dACC) and anterior insula (aINS; Decety et al., 2013a, Fallon et al., 2020, Lamm et al., 2011), core nodes of the salience network which integrate multiple stimuli to coordinate cortical and subcortical resources to respond to motivationally relevant stimuli (Decety, 2011, Harsay et al., 2012, Shackman et al., 2011, Yoder and Decety, 2018).

Moreover, these regions play critical roles in supporting moral decision-making (Decety and Yoder, 2017, Krueger and Hoffman, 2016). Previous work demonstrates that psychopathy, even when not associated with differences in socio-emotional judgments, is related to atypical neural responses within these regions, especially pSTS/TPJ, amygdala, and ventromedial prefrontal cortex (vmPFC), as well as the functional connectivity with dACC and aINS (Harenski et al., 2010, Yoder et al., 2015a, Yoder et al., 2015b). Moreover, individuals with high levels of psychopathic traits appear to not encode the pain of others as personally relevant, though they can make use of this information if it becomes relevant to the task at hand (Yoder et al., 2015a) or if they are imaging themselves suffering (Decety et al., 2013a, Decety et al., 2013b). In fact, recent work suggests that psychopaths do not spontaneously adopt the spatial perspective of others, and that the magnitude of this dysfunction in altercentric interference is correlated with real-world callous behaviors (Drayton et al., 2018).

Given the complexity of the deficits in socioemotional processing and moral cognition in psychopathy, understanding the links between psychopathic traits and moral cognition requires experimental manipulations which focus on specific aspects of socioemotional processing in ecologically meaningful contexts. In particular, there is good evidence that just as individuals with high psychopathic traits “know” moral rules but don’t “care” about them (Cima et al., 2010), such individuals do not attend to socioemotional information in the same way as individuals without psychopathy. Psychopathy is associated with reductions in neural activity and functional connectivity within the salience network when viewing visual depictions of morally laden scenarios, especially when the moral content of the scenarios is relevant to the task (Yoder et al., 2015a). Psychopathy has also been linked to reduced anatomical connectivity of the uncinate fasciculus (Motzkin et al., 2011, Wolf et al., 2015), which links the anterior temporal lobe, including amygdala and aINS, to inferior frontal cortex, including vmPFC.

The first, and to our knowledge, the only fMRI study of psychopathy in female inmates found negative associations between PCL-R scores and hemodynamic responses in right amygdala and rostral ACC during emotional processing, and in the right TPJ specifically during the processing of moral scenarios (Harenski et al., 2014). Recent neuroimaging work in non-incarcerated women identified associations between PCL-R scores and connectivity, both anatomical white matter integrity (Lindner et al., 2017) and functional connectome defined from resting-state data (Lindner et al., 2018). These effects were more particularly pronounced for Factor 2 scores, which reflect the affective/interpersonal dimension of psychopathy, suggesting that this dimension of psychopathy may be most important for understanding the impact of psychopathic traits on neural functioning in female inmates.

The current study was designed to examine socioemotional processing in response to third-party morally laden interactions in female offenders. The emotional expressions of the protagonists were situated in the context of dyadic interactions that were either intentionally harmful or intentionally helpful. Harm and help represent prototypical morally bad and morally good actions and provide a useful platform for examining low-level sociomoral cognition (Yoder et al., 2015a). Based on previous work, it was expected that inmates with higher levels of psychopathy traits would be less likely to find harmful outcomes as salient, leading to reduced hemodynamic response in core nodes of the salience network, particularly dACC, aINS, and amygdala. Moreover, psychopathy was expected to be associated with little to no difference in behavioral responses coincident with a shift towards reliance on prefrontal executive control systems, leading to increased response in dlPFC. This shift was also expected to manifest as reductions in functional connectivity seeded in amygdala and TPJ to other nodes of the salience network and social cognition networks, such as insula, TPJ, and dmPFC.

## 2 Materials and methods

### 2.1 Participants

115 women in a medium-maximum security state prison completed all aspects of the study protocol. Eight participants showed excessive movement in the MRI data (rotation > 3 degrees or translation > 3 mm) and were excluded from analysis. Thus, the final sample consisted of 107 women (M_age_ = 35.0, SD = 8.2, range = 20 – 53). Inclusion criteria were age 18–59 years, female sex (not transitioning), no uncorrectable auditory or visual deficits, ability to speak and understand English, reading level of at least 5th grade, not currently pregnant, no central nervous system disease, no current major medical conditions, no hypertension with complications, no lifetime history of psychotic disorder, no self-reported psychotic disorder (with psychiatric hospitalization) in a first degree relative, no traumatic brain injury with loss of consciousness >10 min, no drug use in last three months (self-report or institution records), and no MRI contraindications such as metal in the body. One participant was ambidextrous, while the remaining 106 were right-handed. 65 (61%) of the women had been convicted of at least one violent crime. The women were compensated for study participation at a rate proportional to the institutional wages for work assignments at their correctional facility, and provided written informed consent. All procedures and materials were approved by the Institutional Review Boards at the University of Chicago and Ethical and Independent Review Services.

Psychopathy was assessed using the Hare Psychopathy Checklist-Revised (Hare, 2003) which was administered by trained research assistants. The PCL-R includes four correlated facets, which can be grouped into two higher order factors (Hare, 2016). Factor 1 captures interpersonal and affective dimensions, while Factor 2 captures developmental, lifestyle, and antisocial aspects of psychopathy. Intelligence Quotient (IQ) was assessed using the vocabulary and matrix reasoning subtests from the Wechsler Adult Intelligence Scale 3rd Edition or the Wechsler Abbreviated Scale of Intelligence 2nd Edition. PCL-R scores showed small but significant negative relationships with age (Spearman’s rho = -0.19, p = 0.049) and IQ (Spearman’s rho = -0.20, p = 0.042). PCL-R scores were not significantly related to conviction for at least one violent crime (Odds Ratio = 1.17, 95% CI [0.79, 1.73], p = 0.435).

### 2.2 Task stimuli

**Fig. 1:**
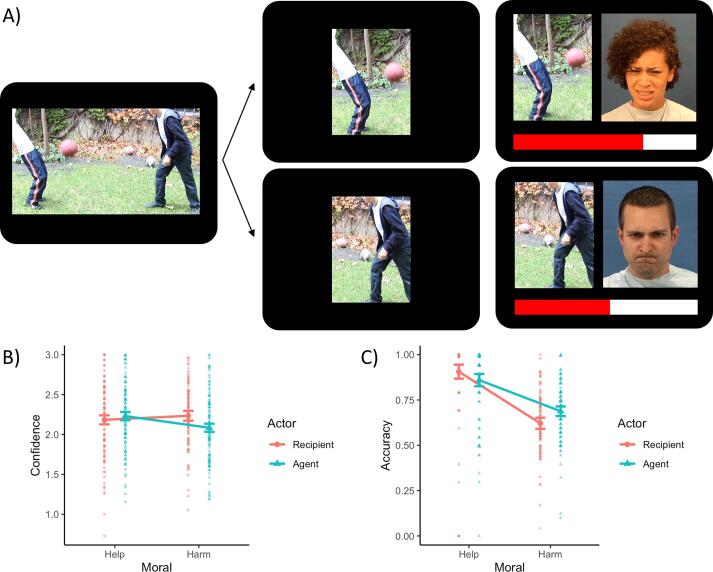
Task schematic and behavioral responses. A) Sample trial of harmful interaction with pain expression for recipient (top) and angry expression for agent (bottom). Below are shown plots for the Actor * Moral interaction for confidence across all responses (B) and accuracy for trials with clear emotion mappings (C).

Participants completed a task previously used in a population of incarcerated males (Decety et al., 2015). In each trial, participants were shown a dyadic interactions depicting either intentional interpersonal harm or intentional assistance. Depictions consisted of three static images presented to create apparent motion (image durations of 1.0, 0.2, and 1.0 s). Following each scenario, participants were shown a cutout of either the recipient of the behavior or the individual who initiated the behavior. After a jittered interval (M = 3 s, SD = 1.2 s), a 2s video clip appeared next to the cut-out and showed a person making one of six expressions: happy, sad, frightened, angry, disgust, or in pain. Importantly, the interaction and cutout did not show a face, so it was possible to counter-balance actors and expressions with recipients or agents (see Fig. 1). After the video ended, the final frame remained on the screen next to the cutout, and participants were asked “Do you think the person felt this way?” Participants indicated their response by pressing a key to stop a red bar which began on the left (“No, not at all”) and moved to the right (“Yes, definitely”). Trials were separated by a jittered interval (M = 3, SD = 1.1 s). Stimuli were presented using the E-Prime 2.0 stimuli presentation suite (Psychology Software Tools, Pittsburgh, PA, USA).

### 2.3 MRI acquisitions and analysis

Function images were acquired using the Mind Research Network 1.5 Tesla Siemens Magnetom Avanto Mobile unit (Washington, DC, USA) which was equipped with a 32-element head coil. Echo-planar images were acquired using a multiband sequence (posterior-to-anterior phase encoding, multiband factor = 12, repetition time/echo time = 350 ms / 39 ms, flip angle = 37 degrees, field of view = 248 × 248 mm, matrix = 70 × 70, voxel size = 3.5 × 3.5 × 3.5 mm^3^). These images were then realigned and motion-corrected using INRIAign (Freire et al., 2002). Rather than performing slice-timing correction at this step, the temporal derivative of each event was included (see below). EPI images were normalized to the EPI MNI template (Calhoun et al., 2017) before smoothing with an 8 mm Gaussian kernel. Images were preprocessed and analyzed using SPM12 (Wellcome Department of Imaging Neuroscience, London, UK) in MATLAB (MathWorks, Natick, MA, USA).

A general linear modeling (GLM) framework was used, where a canonical hemodynamic response function was convolved with a boxcar function representing the onsets and durations of the events of interest. Specifically, the onsets of each scenario through the end of the third picture, and the onset of each decision phase, beginning at the onset of the actor cutout through the response. This created six trial regressors: HarmScene, HelpScene, IdentifyAgentHarm, IdentifyRecipientHarm, IdentifyAgentHelp, IdentifyRecipientHelp. Temporal derivatives were also modeled for each event. The beta image pairs for each modeled event amplitude and temporal derivative were combined into a single magnitude image which was then passed to the second-level analysis (Calhoun et al., 2004). Six movement parameters were entered as nuisance regressors.

Second-level contrasts were derived by combining first-level contrast estimates. Psychopathy scores were modeled using either total PCL-R, or Factor 1 and Factor 2. For each, mean-centered age in months and IQ were entered as covariates of no interest. For group-based analysis, participants with PCL-R scores of 30 or above (n = 24) were categorized as high psychopathy, consistent with the “diagnostic” cutoff proposed by Hare (2003), and scores of 20 or below (n = 45) were categorized as low. Functional connectivity was assessed by modeling a psychophysiological interaction between the task contrasts and mean signal extracted from an anatomically defined right amygdala mask and a 10 mm radius sphere placed in rTPJ (MNI ×  = 52, y = -54, z = 16) based on previous work investigating rTPJ connectivity during socioemotional processing (Yoder et al., 2015a, Yoder and Decety, 2014a). Images were thresholded to achieve family-wise error corrected p < 0.05, determined using the first-level residual images to estimate smoothness for 3dClustSim (Cox, 1996).

### 2.4 Behavioral data analysis

Two separate measures were extracted from the behavioral responses (Decety et al., 2015). First, the midpoint was subtracted from the responses and the absolute value was taken to create a “confidence” measure, ranging from 0, the midpoint, to 3, the extreme end of the scale. Accuracy was evaluated by limiting analyses to those trials where there was a natural congruence between an individual’s emotion and the situation: happy expressions for either individuals following a helpful interaction, angry expressions for the perpetrators of harmful interactions, and pain or sad expressions for victims of harmful interactions.

Behavioral data were analyzed using complementary approaches in R (version 4.0.2, R Core Team, 2015). First, repeated-measures analysis of variance (ANOVA) modeled confidence and accuracy in a 2 (Morality: Harm|Help) × 2 (Actor: Agent|Recipient) basic model using the ‘afex’ package (Singmann et al., 2020) with pairwise comparisons interrogated with the ‘emmeans’ package (Lenth, 2020). Once fit to the full model, another model specifically examined high and low psychopathy groups. A complementary multilevel linear modeling (MLM) approach, as implemented in the ‘lme4′ package (Bates et al., 2015), regressed behavioral responses on Morality and Actor, with participant modeled using a random intercept. Psychopathy was modeled continuously using PCL-R scores.

## 3 Results

The ANOVA for confidence identified a marginal main effect of Actor (F(1,106, F = 3.92, η^2^_G_=0.003, p = 0.050) and a significant Moral * Actor interaction (F(1,106), F = 15.89, η^2^_G_=0.013, p < 0.001; Fig. 1B). Tukey’s comparisons revealed higher confidence for recipients than agents in harmful interactions (p = 0.002) and higher confidence for helpful agents than harmful agents (p < 0.001). These effects remained the same when limiting the analysis to only the high and low psychopathy groups. No Group effects were significant (all p > 0.3). Modeling psychopathy scores continuously also did not identify any significant effects (all p > 0.2), and including psychopathy scores did not significantly improve model fit over a model with just Moral and Actor terms (*Χ^2^*(4) = 6.17, p = 0.187).

Accuracy for the subset of trials with clear emotional mapping revealed a significant main effect of Moral (F(1, 106) = 190.53, η^2^_G_ = 0.278, p < 0.001) and a Moral * Actor interaction (F(1, 106) = 9.75, η^2^_G_ = 0.023, p = 0.002; Fig. 1C). All pairwise comparisons were significant (largest p = 0.035 for recipients of harm compared to agents of harm). Accuracy was highest for recipients of help, then agents of help, agents and harm, and recipients of harm had the lowest accuracy. As with confidence, restricting the analysis to only high and low psychopathy individuals produced similar results, though the pairwise difference between agents and recipients of harm became non-significant (p = 0.192). No Group effects were significant (all p > 0.3). Modeling psychopathy continuously in an MLM framework produced similar effects, with psychopathy scores producing no significant behavioral effects (all p > 0.6) and psychopathy score not improving explanatory power of the model (*Χ^2^*(4) = 0.69, p = 0.953).

**Fig. 2:**
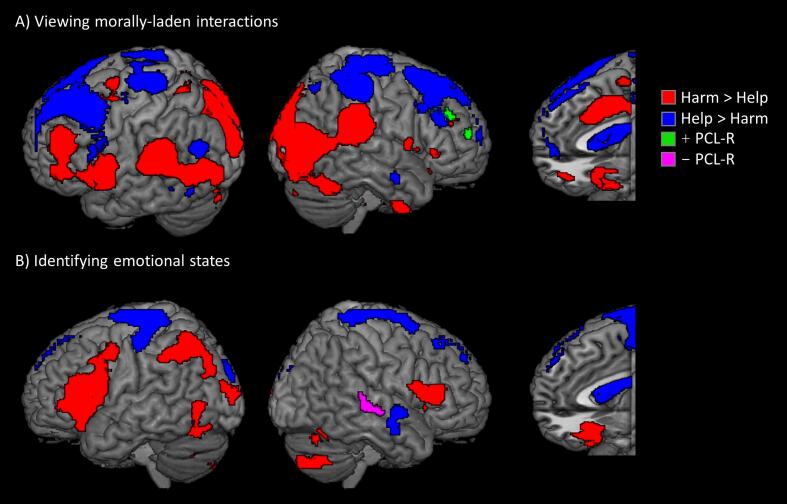
Whole-brain results for morally laden content during task phases. Regions more sensitive to harmful (red) or helpful (blue) interactions during the viewing phase (A) or emotion identification phase (B). Also shown are regions identified by the Harm-Help contrast as having a positive association (green) or negative association (violet) with PCL-R scores. All regions significant at FWEp < 0.05 (height = 0.005, extent = 100). (For interpretation of the references to colour in this figure legend, the reader is referred to the web version of this article.)

Viewing harmful social interactions compared to helpful social interactions elicited increased hemodynamic response throughout visual cortex, much of the social decision-making network, including pSTS/TPJ, and the core nodes of the salience network – i.e. dACC, aINS (Fig. 2A, Table S1). In contrast, helpful actions were associated with greater signal in bilateral caudate, vmPFC, and dlPFC. PCL-R scores were associated with greater responses in right dlPFC when viewing harmful compared to helpful interactions. Factor 1 and Factor 2 scores were not uniquely associated with any significant clusters.

During the emotion identification phase, decisions about harmful interactions, compared to helpful interactions, elicited greater response in lateral occipital cortex, fusiform gyrus, and bilateral inferior frontal gyrus (IFG) extending into aINS and left inferior parietal (Fig. 2B; Table S1). Decisions about helpful interactions were associated with greater response in primary visual cortex, left precentral gyrus, right aINS, SMA, and right caudate body. Combined with the viewing phase, these main effects replicate previous whole-brain results using the same task in a sample of incarcerated males (Decety et al., 2015), with the exception of rTPJ, in which activation was not observed during the evaluation phase in this study. PCL-R scores were associated with reduced response in right STS when identifying the emotions of individuals involved in harmful compared to helpful interactions. Factor 1 scores were specifically associated with reduced signal in right STS. No clusters showed any significant associations with Factor 2 scores.

**Fig. 3:**
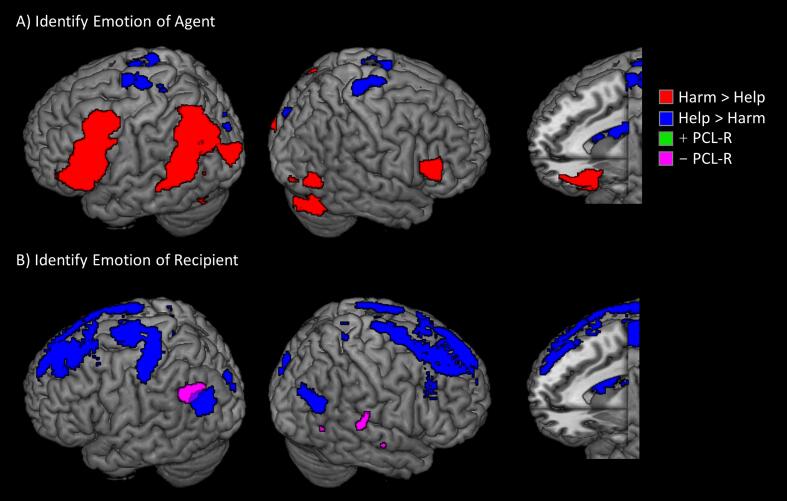
Whole-brain results for identifying emotional stats of different individuals. Regions more sensitive to harmful (red) or helpful (blue) interactions when evaluating the emotional state of agents who initiated actions (A) or recipients of actions (B). Also shown are regions identified by the Harm-Help contrast as having a positive association (green) or negative association (violet) with PCL-R scores. All regions significant at FWEp < 0.05 (height = 0.005, extent = 100). (For interpretation of the references to colour in this figure legend, the reader is referred to the web version of this article.)

When evaluating the emotions of protagonists who initiated harmful compared to helpful interactions, greater neuro-hemodynamic response was observed in bilateral IFG and left aINS, dlPFC, and TPJ (Fig. 3A; Table S2). Identifying the emotional state of actors in helpful scenarios elicited greater signal in cuneus, SMA, precentral gyrus and caudate. When identifying the emotions of recipients of helpful compared to harmful interactions, increased signal was observed in cuneus, SMA, caudate body, and bilateral dlPFC (Fig. 3B). No regions showed greater response when identifying recipients of harm compared to help. PCL-R scores showed no significant associations when identifying the emotions of agents, but were associated with reduced signal in bilateral pSTS when identifying the emotional state of the recipients of harm compared to help (Fig. 3B). Factor 1 scores were not significantly associated with either contrast, but Factor 2 scores were significantly associated with reduced response in right pSTS, caudate, and ACC (Table S2).

**Fig. 4:**
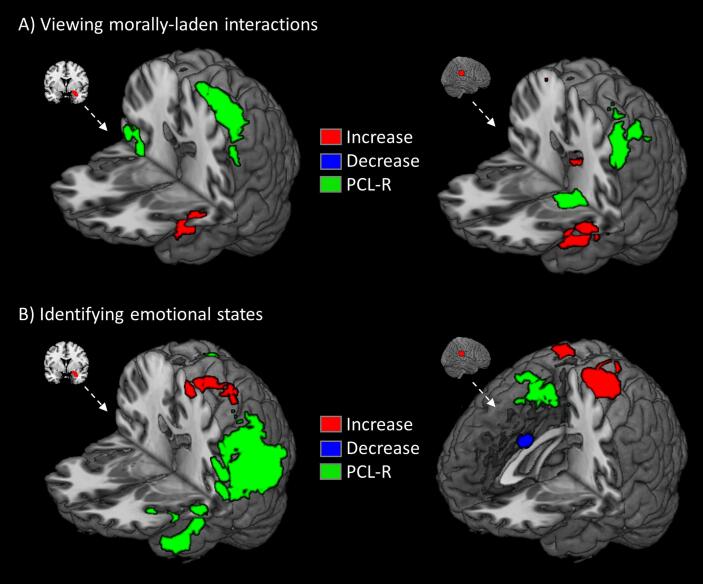
Whole-brain functional connectivity results for task phases. Regions show increased (red) or decreased (blue) connectivity with right amygdala (left) or TPJ (right) during the view phase (A) or emotion identification phase (B). Regions where connectivity was negatively associated with PCL-R scores are shown in green. All regions significant at FWEp < 0.05 (height = 0.005, extent = 100). (For interpretation of the references to colour in this figure legend, the reader is referred to the web version of this article.)

When viewing harmful compared to helpful interactions, right amygdala demonstrated increased functional connectivity with left aINS (Fig. 4; Table S3). The rTPJ seed showed increased neuronal coupling with left medial temporal areas, caudate and right medial and superior frontal cortex (Fig. 4; Table S4). Right TPJ showed increased connectivity with an overlapping cluster in left parietal, as well as increased connectivity with precuneus and right parietal. When viewing harmful compared to helpful interactions, PCL-R scores were associated with reduced functional connectivity from amygdala to left parietal and right temporal cortex, and with reduced connectivity from TPJ to left parietal and parahippocampal gyrus (Fig. 4A).

While identifying emotions for harmful compared to helpful interactions (Fig. 4B), right amygdala demonstrated increased connectivity with a cluster in left parietal cortex extending to TPJ and into anterior insula. Right TPJ also showed decreased connectivity with right fusiform gyrus and dACC. PCL-R scores were associated with decreased amygdala connectivity with precuneus and bilateral pSTS, extending into left insula. Connectivity between rTPJ and dACC/SMA was also negatively related to PCL-R scores.

**Fig. 5:**
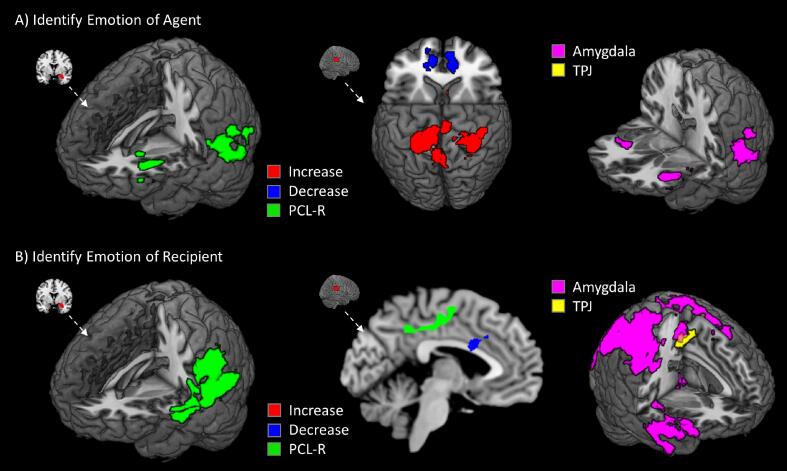
Whole-brain functional connectivity results for different actors. Regions show increased (red) or decreased (blue) connectivity with right amygdala (left) or TPJ (right) when identifying agents who initiated actions (A) or recipients (B). Regions where connectivity was negatively associated with PCL-R scores are shown in green. At right are shown regions where Factor 2 scores were negatively associated with functional connectivity seeded in amygdala (violet) or TPJ (yellow). All regions significant at FWEp < 0.05 (height = 0.005, extent = 100). (For interpretation of the references to colour in this figure legend, the reader is referred to the web version of this article.)

Identifying the emotions of agents who initiated actions (Fig. 5A; Table S6) elicited reduced connectivity from rTPJ to vmPFC and increased connectivity with caudate and bilateral postcentral gyri. Specifically focusing on identifying the emotional state of the agent of the interaction, PCL-R scores were negatively related to connectivity between right amygdala and left aINS, TPJ, IFG, precuneus (Table S5). PCL-R scores were not related to functional connectivity seeded in rTPJ during identification of agent emotions. Factor 2 was specifically associated with decreased functional connectivity between amygdala and left pSTS and bilateral aINS.

For recipients (Fig. 5B), TPJ showed greater connectivity with dACC/SMA. No regions showed significant functional connectivity increases or decreases with right amygdala when examining harmful compared to helpful interaction specifically for the actor or specifically for the recipient. PCL-R scores were significantly related to reduced connectivity between right amygdala and left temporal regions, including pSTS, and reduced connectivity between right TPJ and SMA. Factor 2 scores were associated with decreased connectivity between both amygdala and TPJ with SMA, and between amygdala and right aINS, inferior frontal gyrus, and inferior parietal cortex.

## 4 Discussion

The goal of this study was to examine the impact of psychopathic traits on behavioral and neuro-hemodynamic measures of socioemotional understanding of morally laden interactions in adult female offenders. Overall, and as predicted, psychopathy, whether viewed as a dichotomous variable or a continuous trait, was not related to differences in behavioral responses. Confidence ratings and behavioral accuracy were not significantly related to PCL-R scores, and the high and low psychopathy groups did not significantly differ on either measure. Contrary to predictions, psychopathy was not significantly related to reduced responses in amygdala or dACC during either third-party evaluation or identification phases. While women overall did demonstrate greater dACC response when viewing harmful compared to helpful interactions, no amygdala response was detected in either phase. However, psychopathy scores were significantly related to increased hemodynamic response in rdlPFC when viewing harmful scenarios and widespread decreases in functional connectivity seeded in right amygdala and right TPJ. Overall, these results suggest that the nodes of the salience and social cognition networks respond similarly in female psychopaths, but the networks are largely disconnected in comparison to inmates with low levels of psychopathic traits.

The whole-brain results for viewing harmful compared to helpful interactions is consistent with a large body of work in moral neuroscience and third-party evaluations (Eres et al., 2018, Krueger and Hoffman, 2016), with regions important for theory of mind and saliency, especially bilateral TPJ, insula, and dACC/SMA showing greater response to intentional harm (Decety and Yoder, 2017, Yoder and Decety, 2014a). In contrast, core regions of the reward circuitry, namely vmPFC and caudate, as well as dlPFC demonstrated greater response when observing helpful interactions (Decety and Porges, 2011).

Right dlPFC response directly replicates previous moral judgment work using similar stimuli in undergraduates and inmates (Yoder et al., 2015a, Yoder and Decety, 2014a, Yoder and Decety, 2014b). The dlPFC was the only region showing significant associations with PCL-R total score (Fig. 2A). This result fits with the notion that individuals with high levels of psychopathic traits rely on prefrontal recruitment in order to maintain similar behavior responses (Glenn et al., 2009b, Yoder et al., 2015a).

When identifying emotions of individuals involved in harmful compared to helpful interactions, psychopathic traits were associated with reduced response in a region of right STS extending into deep pSTS/TPJ (Fig. 2B). Response in pSTS is reliably implicated when inferring mental states of others in pain (Lamm et al., 2011), but pSTS also plays important integrative roles for incorporating mental state information into social decision-making contexts (Carter and Huettel, 2013, Yoder and Decety, 2018). Reduced response in this region was specifically associated with Factor 1, but not Factor 2. Thus, reduced TPJ response here suggests that women with higher levels of psychopathic traits, particularly the interpersonal-affective dimension of callousness, may rely less on others' mental states when attempting to label emotional expressions of others. Future work could clarify this relationship by examining emotional accuracy while varying the amount of mental state information that is available and testing whether Factor scores are associated with specific decreases in performance.

When identifying the emotion of the recipient of an interaction (Harm – Help), PCL-R scores were associated with reduced response in bilateral pSTS (Fig. 3B). Interestingly, whereas pSTS response when identifying emotions generally was associated with Factor 1 scores, Factor 2 scores were associated with reduced pSTS response specifically for identifying the emotions of recipients (Table S2). This dissociation between Factor 1 and 2 suggests that the Interpersonal/Affective and Developmental/Lifestyle/Antisocial dimensions of psychopathy may differentially impact use of mental state information when evaluating the emotional states of others in general or specifically of victims. The lack of an association between psychopathic traits and amygdala or dACC response is surprisingly. However, this result could be a consequence of directly comparing harmful and helpful interactions, rather than including a neutral condition. Further work is required to directly test this possibility.

The impact of psychopathic traits was more robust when examining functional connectivity. When viewing morally laden scenarios (Harm – Help), PCL-R scores were associated with reduced functional connectivity to left inferior parietal cortex for both the amygdala and TPJ seeds (Fig. 4). Moreover, the amygdala seed revealed psychopathy-linked reduced connectivity between right amygdala and right TPJ. These alterations in functional connectivity are consistent with recent reports of disrupted functional networks association with higher psychopathic traits in incarcerated males (Espinoza et al., 2018, Tillem et al., 2019). Thus, psychopathy appears to alter connectivity within the social cognition network when inmates viewed morally laden images.

During the emotion identification phase, PCL-R scores predicted reduced connectivity between right amygdala and left TPJ. This effect remained regardless of whether the trial was focused on the agent or the recipient, suggesting that for individuals with high levels of psychopathic traits the usually aversive salience of interpersonal harm is disconnected from mental state representations (Buckholtz and Marois, 2012). For agents, PCL-R was also related to reduced connectivity to aINS. This fits with previous work demonstrating reduced aINS response during face processing among adolescent females with conduct disorder (Fairchild et al., 2014). This is particularly important given the role of the insula in salience processing and signaling motivationally relevant information (Harsay et al., 2012, Krueger and Hoffman, 2016). The negative relationship between PCL-R and amygdala-insula coupling suggests that individuals with high levels of psychopathic traits don’t encode violent others as personally salient, potentially because they don’t view others behaving antisocially as unexpected. This would be consistent with studies linking increased psychopathic traits in the general population to reduced amygdala connectivity (e.g., Dotterer et al., 2020, Waller et al., 2019, Yoder et al., 2015b). However, future studies could directly test this effect using violation of expectation paradigms.

Interestingly, while Factor 1 scores were not significantly associated with any changes in functional connectivity during the recipient identification phase, higher Factor 2 scores were associated with reductions in connectivity, specifically to dACC/SMA. Some previous work with undergraduate and incarcerated males found that callous-unemotional traits were specifically linked to reduced connectivity with dACC and right amygdala (Yoder et al., 2015b, Yoder et al., 2015a). Moreover, large-scale investigations of functional connectivity in incarcerated males has linked Factor 1, rather than Factor 2, to altered network connectivity, particularly with the salience network (Espinoza et al., 2018, Thijssen and Kiehl, 2017). Thus, the somewhat surprising link with Factor 2 in the current study suggests that the dimensions of psychopathy may impact different neural systems in women and men.

## 5 Conclusion

Overall, the results of our study replicate previous work demonstrating links between higher levels of psychopathic traits and widespread decreases in functional connectivity seeded in amygdala and TPJ during socioemotional processing and decision-making. Importantly, the current study extends these findings to incarcerated females with psychopathic traits, a population that is severely understudied. Much previous work has highlighted specific links between psychopathy and reduced hemodynamic response and connectivity within neural networks anchored by amygdala, dACC, and aINS. However, these results provide important preliminary evidence that the antisocial dimension of psychopathy is more important than the affective/interpersonal dimension for explaining this effect in women.

## CRediT authorship contribution statement

**Keith J. Yoder:** Methodology, Software, Writing - original draft, Writing - review & editing, Visualization. **Carla Harenski:** Data curation, Validation, Writing - original draft. **Kent A. Kiehl:** Data curation, Project administration, Writing - original draft. **Jean Decety:** Conceptualization, Methodology, Writing - original draft, Writing - review & editing, Funding acquisition.

## Declaration of Competing Interest

The authors declare that they have no known competing financial interests or personal relationships that could have appeared to influence the work reported in this paper.
