# Supplementary material for: Neural responses to morally laden interactions in female inmates with psychopathy
Source: Neuroimage Clin. 2021 Mar 27;30:102645. doi: 10.1016/j.nicl.2021.102645 (PMC8045040; doi:10.1016/j.nicl.2021.102645)
Supplement: Supplementary data 1 [file mmc1.docx]

**Table S1. Whole-Brain Results for View and Identify Task Phases.**

|  |  | MNI coordinates | | |  | |  |
| --- | --- | --- | --- | --- | --- | --- | --- |
| Contrast | Region | X | Y | Z | | k | T |
| View (Harm > Help) | |  |  |  | |  |  |
|  | Visual Cortex | 21 | -85 | -11 | | 6548 | 11.43 |
|  | R pSTS/TPJ | 54 | -34 | 22 | |  | 10.95 |
|  | L pSTS/TPJ | -54 | -37 | 22 | |  | 10.87 |
|  | L IFG | -54 | 5 | 10 | | 1027 | 7.33 |
|  | L dlPFC | -33 | 38 | 25 | |  | 6.54 |
|  | L insula | -27 | 17 | -5 | |  | 4.37 |
|  | Midcingulate | -12 | -25 | 37 | | 1143 | 7.17 |
|  | dACC | -6 | 8 | 40 | |  | 5.50 |
|  | R Temporal Pole | 30 | 5 | -23 | | 136 | 4.16 |
|  | Angular Gyrus | -51 | -70 | 25 | | 100 | -4.67 |
|  | Superior Frontal Gyrus | -12 | 38 | 55 | | 4026 | -8.64 |
|  | vmPFC | 3 | 59 | -5 | |  | -4.21 |
|  | Caudate | -3 | 8 | 10 | |  | -8.50 |
|  | R Inferior Parietal | 45 | -37 | 52 | |  | -6.92 |
| PCL-R | |  |  |  | |  |  |
|  | R dlPFC | 42 | 38 | 31 | | 111 | 3.68 |
|  | |  |  |  | |  |  |
| Identify (Harm > Help) | |  |  |  | |  |  |
|  | L Fusiform | -30 | -64 | -17 | | 451 | 5.99 |
|  | L aINS | -33 | 20 | -2 | | 1111 | 5.90 |
|  | L IFG | -51 | 11 | 13 | |  | 5.34 |
|  | L Inferior Parietal | -39 | -55 | 37 | | 851 | 5.17 |
|  | L Visual Cortex | -30 | -97 | 10 | |  | 4.13 |
|  | Cerebellum | 33 | -73 | -50 | | 305 | 4.89 |
|  | R IFG | 48 | 29 | 7 | | 248 | 4.45 |
|  | R aINS | 48 | -1 | -5 | | 190 | -3.79 |
|  | Superior Frontal | 0 | 53 | 40 | | 221 | -3.85 |
|  | Lingual Gyrus | 6 | -64 | -2 | | 183 | -4.04 |
|  | L Precentral Gyrus | -18 | -19 | 73 | | 1662 | -4.99 |
|  | SMA | 0 | -19 | 70 | |  | -4.53 |
|  | Visual Cortex | -15 | -91 | 28 | | 400 | -5.21 |
|  | Caudate | 3 | 17 | 10 | | 769 | -6.05 |
| PCL-R | |  |  |  | |  |  |
|  | R pSTS | 33 | -34 | 4 | | 295 | -3.92 |
| Factor 1 | |  |  |  | |  |  |
|  | R pSTS | 33 | -49 | 10 | | 186 | -4.01 |
| aINS: anterior insula; dACC: dorsal anterior cingulate cortex; dlPFC: dorsolateral prefrontal cortex; IFG: inferior frontal gyrus; pSTS: posterior superior temporal sulcus. TPJ: temporo-parietal junction; SMA: supplementary motor area. Significant at FWEp < .05 (p = .005, k = 100) | | | | | | | |

**Table S2. Whole-Brain Results for Emotional Identification of Agents and Recipients**

|  |  | MNI coordinates | | |  |  |
| --- | --- | --- | --- | --- | --- | --- |
| Contrast | Region | X | Y | Z | k | T |
| Identify Agent (Harm > Help) | |  |  |  |  |  |
|  | L IFG | -48 | 23 | 1 | 1385 | 6.49 |
|  | L aINS | -33 | 20 | -2 |  | 5.11 |
|  | L Angular Gyrus | -36 | -61 | 34 | 1770 | 5.27 |
|  | Cerebellum | 15 | -82 | -26 | 397 | 4.41 |
|  | R IFG | 51 | 26 | 7 | 143 | 4.20 |
|  | L Fusiform Gyrus | -33 | -61 | -17 | 183 | 3.90 |
|  | R Occipital | 42 | -73 | -5 | 130 | 3.60 |
|  | SMA | -12 | -10 | 70 | 226 | -3.89 |
|  | R Precentral Gyrus | 21 | -31 | 70 | 135 | -4.03 |
|  | Cuneus | -3 | -85 | 25 | 160 | -4.89 |
|  | Caudate | 6 | -7 | 19 | 399 | -4.91 |
|  | |  |  |  |  |  |
| Identify Recipient (Harm > Help) | |  |  |  |  |  |
|  | Cuneus | -12 | -91 | 28 | 220 | -3.86 |
|  | R Angular Gyrus | 42 | -61 | 22 | 156 | -4.09 |
|  | L Angular Gyrus | -48 | -73 | 25 | 174 | -4.53 |
|  | Caudate | 6 | 14 | 13 | 301 | -5.73 |
|  | Superior Frontal Gyrus | 0 | 56 | 37 | 1640 | -6.01 |
|  | SMA | 0 | -16 | 61 |  | -3.46 |
| PCL-R | |  |  |  |  |  |
|  | R pSTS | 36 | -31 | -2 | 484 | -4.30 |
|  | L pSTS | -48 | -61 | 31 | 128 | -4.16 |
| Factor 2 | |  |  |  |  |  |
|  | R pSTS | 45 | -43 | -2 | 118 | -3.86 |
|  | R Caudate | 27 | 8 | 16 | 185 | -4.38 |
|  | ACC | 0 | 29 | 10 | 135 | -3.85 |
| ACC: anterior cingulate cortex; aINS: anterior insula; IFG: inferior frontal gyrus; pSTS: posterior superior temporal sulcus. TPJ: temporo-parietal junction; SMA: supplementary motor area. Significant at FWEp < .05 (p = .005, k = 100) | | | | | | |

**Table S3. Right Amygdala Functional Connectivity Whole-Brain Results for Task Phases**

|  |  | MNI coordinates | | |  |  |
| --- | --- | --- | --- | --- | --- | --- |
| Contrast | Region | X | Y | Z | k | T |
| View (Harm > Help) | |  |  |  |  |  |
|  | L aINS | -36 | 2 | -23 | 144 | 4.31 |
| PCL-R | |  |  |  |  |  |
|  | R pSTS | 45 | -22 | -2 | 140 | -3.95 |
|  | L Postcentral | -36 | -34 | 61 | 204 | -4.07 |
| Factor 1 | |  |  |  |  |  |
|  | dmPFC | 0 | 50 | 31 | 121 | -4.33 |
|  |  |  |  |  |  |  |
| Identify (Harm > Help) | |  |  |  |  |  |
|  | L Postcentral | -18 | -16 | 70 | 129 | 3.89 |
| PCL-R | |  |  |  |  |  |
|  | L pSTS | -63 | -40 | 16 | 1790 | -5.64 |
|  | L Insula | -45 | -4 | 1 |  | -4.35 |
|  | L Temporal Pole | -42 | 11 | -32 |  | -4.90 |
|  | R pSTS | 54 | -52 | 4 | 416 | -4.18 |
|  | Precuneus | 9 | -58 | 64 | 124 | -3.96 |
| Factor 1 | |  |  |  |  |  |
|  | L TPJ | -63 | -37 | 13 | 146 | -4.16 |
| Factor 2 | |  |  |  |  |  |
|  | L Temporal Pole | -42 | 11 | -32 | 7997 | -5.60 |
|  | L aINS | -33 | 26 | -8 |  | -4.29 |
|  | L TPJ | -54 | -49 | 19 |  | -4.81 |
|  | R Temporal Pole | 45 | 11 | -26 | 117 | -3.77 |
|  | R aINS | 48 | 26 | -8 | 288 | -4.09 |
|  | R TPJ | 60 | -49 | 7 | 172 | -4.30 |
|  | dACC | -9 | 17 | 28 | 126 | -3.79 |
| aINS: anterior insula; dACC: dorsal anterior cingulate cortex; dmPFC: dorsomedial prefrontal cortex; pSTS: posterior superior temporal sulcus. TPJ: temporo-parietal junction. Significant at FWEp < .05 (p = .005, k = 100) | | | | | | |

**Table S4. Right TPJ Functional Connectivity Whole-Brain Results for Task Phases**

|  |  | MNI coordinates | | |  |  |
| --- | --- | --- | --- | --- | --- | --- |
| Contrast | Region | X | Y | Z | k | T |
| View (Harm > Help) | |  |  |  |  |  |
|  | Temporal Pole | -48 | 2 | -11 | 124 | 4.23 |
|  | Caudate | -3 | 11 | 10 | 306 | 5.43 |
|  | R Medial Frontal | 12 | 59 | 34 | 410 | 4.97 |
|  | R Superior Frontal | 30 | 5 | 64 | 363 | 4.00 |
| PCL-R | |  |  |  |  |  |
|  | Parahippocampal | -15 | -10 | -8 | 412 | -4.70 |
|  | L Inferior Parietal | -45 | -31 | 43 | 189 | -4.15 |
| Factor 2 | |  |  |  |  |  |
|  | L Superior Parietal | -36 | -37 | 58 | 523 | -4.75 |
|  | R Precentral | 21 | -22 | 67 | 535 | -4.07 |
|  | SMA | 3 | -1 | 58 |  | -3.33 |
|  | |  |  |  |  |  |
| Identify (Harm > Help) | |  |  |  |  |  |
|  | R Fusiform | 21 | -46 | -5 | 120 | -4.37 |
|  | dACC | -12 | 11 | 31 | 167 | -4.37 |
|  | R Poscentral | 33 | -37 | 67 | 143 | 4.60 |
|  | L Postcentral | -18 | -37 | 67 | 230 | 4.63 |
|  | Precuneus | 0 | -49 | 67 |  | 4.11 |
| PCL-R | |  |  |  |  |  |
|  | Cerebellum | -6 | -52 | -11 | 183 | -3.82 |
|  | SMA | 24 | -1 | 52 | 247 | -4.24 |
| Factor 2 | |  |  |  |  |  |
|  | L Hippocampus | -30 | -31 | -5 | 137 | -4.80 |
|  | L Superior Parietal | -30 | -31 | 55 | 159 | -4.09 |
|  | dACC/SMA | -18 | 8 | 43 | 218 | 4.74 |
| dACC: dorsal anterior cingulate cortex; SMA: supplementary motor areaSignificant at FWEp < .05 (p = .005, k = 100) | | | | | | |

**Table S5. Right Amygdala Functional Connectivity for Agent and Recipient Evaluations**

|  |  | MNI coordinates | | |  |  |
| --- | --- | --- | --- | --- | --- | --- |
| Contrast | Region | X | Y | Z | k | T |
| Identify Agent (Harm > Help) | | |  |  |  |  |
|  |  |  |  |  |  |  |
| PCL-R |  |  |  |  |  |  |
|  | L IFG | -48 | 2 | 43 | 409 | -4.15 |
|  | L aINS | -39 | 14 | -5 |  | -3.24 |
|  | R pSTS/TPJ | -57 | -61 | -2 | 277 | -3.66 |
|  | Precuneus | 24 | -61 | 16 | 379 | -4.29 |
|  |  |  |  |  |  |  |
| Identify Recipient (Harm > Help) | | |  |  |  |  |
|  |  |  |  |  |  |  |
| PCL-R |  |  |  |  |  |  |
|  | L Superior Temporal | -60 | -13 | 4 | 654 | -4.22 |
| Factor 2 | |  |  |  |  |  |
|  | R Inferior Temporal | 42 | -7 | -41 | 196 | -4.50 |
|  | L pSTS/TPJ | -60 | -19 | 37 | 3681 | -5.25 |
|  | SMA | -3 | -22 | 52 |  | -3.77 |
|  | R Postcentral | 45 | -28 | 58 |  | -3.92 |
|  | Precuneus | 9 | -55 | 61 |  | -4.23 |
|  | Cerebellum | -21 | -49 | -17 | 270 | -4.05 |
|  | R Superior Occipital | 33 | -76 | 43 | 123 | -3.95 |
| aINS: anterior insula; IFG: inferior frontal gyrus; pSTS: posterior superior temporal sulcus. TPJ: temporo-parietal junction. Significant at FWEp < .05 (p = .005, k = 100) | | | | | | |

**Table S6. Right TPJ Functional Connectivity for Agent and Recipient Evaluations**

|  |  | | MNI coordinates | | |  |  |
| --- | --- | --- | --- | --- | --- | --- | --- |
| Contrast | Region | | X | Y | Z | k | T |
| Identify Agent (Harm > Help) | | | |  |  |  |  |
|  | | vmPFC | -9 | 50 | 10 | 474 | -3.98 |
|  | | Caudate | 3 | 5 | 13 | 140 | 4.12 |
|  | | R Postcentral | 30 | -34 | 67 | 179 | 4.10 |
|  | | L Postcentral | -24 | -34 | 67 | 285 | 4.29 |
|  | |  |  |  |  |  |  |
| Identify Recipient (Harm > Help) | | | |  |  |  |  |
|  | | dACC | 0 | 11 | 28 | 129 | -4.22 |
| PCL-R | |  |  |  |  |  |  |
|  | | SMA | 0 | -34 | 43 | 226 | -3.42 |
| Factor 2 | | |  |  |  |  |  |
|  | | L Hippocampus | -33 | -28 | -5 | 128 | -4.27 |
|  | | SMA | -27 | -25 | 52 | 129 | -3.69 |
| dACC: dorsal anterior cingulate cortex; SMA: supplementary motor area; vmPFC: ventromedial prefrontal cortex. Significant at FWEp < .05 (p = .005, k = 100) | | | | | | | |
